# Prevalence and patterns of testing for anaemia in primary care in England: a cohort study using an electronic health records database

**DOI:** 10.3399/BJGP.2024.0336

**Published:** 2025-02-25

**Authors:** Margaret Smith, Cynthia Wright Drakesmith, Sarah Haynes, Suzanne Maynard, Akshay Shah, Noemi BA Roy, Joseph Jonathan Lee, Katja Maurer, Simon J Stanworth, Clare R Bankhead

**Affiliations:** Nuffield Department of Primary Care Health Sciences, University of Oxford, Oxford.; Nuffield Department of Primary Care Health Sciences, University of Oxford, Oxford.; John Radcliffe Hospital, University of Oxford, Oxford.; Radcliffe Department of Medicine, John Radcliffe Hospital, University of Oxford, Oxford.; Nuffield Department of Clinical Neurosciences and NIHR Blood and Transplant Research Unit in Data Driven Transfusion Practice, Radcliffe Department of Medicine, University of Oxford, Oxford.; Department of Haematology, Oxford University Hospitals NHS Foundation Trust, Oxford, and Radcliffe Department of Medicine, University of Oxford, Oxford.; Nuffield Department of Primary Care Health Sciences, University of Oxford, Oxford.; Nuffield Department of Primary Care Health Sciences, University of Oxford, Oxford.; NIHR Blood and Transplant Research Unit in Data Driven Transfusion Practice, Radcliffe Department of Medicine, University of Oxford, Oxford; consultant haematologist, Department of Haematology/Transfusion Medicine, NHS Blood and Transplant, John Radcliffe Hospital, Oxford, and Oxford University Hospitals NHS Foundation Trust, Oxford; Nuffield Department of Primary Care Health Sciences, University of Oxford, Oxford.

**Keywords:** anaemia, epidemiology, ferritins, haemoglobin, mean corpuscular volume, primary health care

## Abstract

**Background:**

Despite epidemiological data on anaemia being available on a global scale, the prevalence of anaemia in the UK is not well described.

**Aim:**

To describe anaemia prevalence and testing patterns for haemoglobin and other blood parameters.

**Design and setting:**

This study was a descriptive population-based cohort study using data drawn from the Clinical Practice Research Datalink Aurum database in 2019.

**Method:**

Demographic data were extracted for each person who was registered at their current practice during 2019, including linked data on Index of Multiple Deprivation. Anaemia prevalence in 2019 was calculated based on World Health Organization-specified age and gender thresholds for haemoglobin. Anaemia was classified based on mean corpuscular volume and ferritin. People with anaemia were followed up for up to 1 year to investigate longitudinal testing patterns for haemoglobin.

**Results:**

The cohort contained 14 million people. Anaemia prevalence in England in 2019 was 4.1% (583 847/14 207 841) (5.1% [363 438/7 121 614] females and 3.1% [220 409/7 086 227] males). Prevalence was higher in people aged >65 years, people of Black and Asian ethnicities, and people living in areas with higher social deprivation. Only half of people with anaemia and a mean corpuscular volume of ≤100 fL had an accompanying ferritin value recorded. About half of people with anaemia had a follow-up haemoglobin test within 1 year, most of which still indicated anaemia.

**Conclusion:**

Anaemia is prevalent in the UK with large disparities between levels of demographic variables. Investigation and follow-up of anaemia is suboptimal in many patients. Health interventions aimed at improving anaemia investigation and treatment are needed, particularly in the most at-risk groups.

## Introduction

Anaemia is a global health problem. Although anaemia prevalence is highest in low-and middle-income countries, the Global Burden of Disease Study estimated that its prevalence in Western Europe in 2021 was 6.0%.^1^ The aetiology of anaemia is multifactorial; however, iron deficiency (where there are insufficient stores of iron in the body or an inability to utilise them) remains the commonest cause and is potentially readily treated.^2^ Other causes of anaemia in the general population include deficiencies of other nutrients (usually B12 or folate), anaemia of chronic disease, and inherited causes. There is less information on the relative proportion of these different aetiologies.^3^

Anaemia contributes significantly to the morbidity and mortality of a population.^1^^,^^4^^,^^5^ Appropriate diagnosis and treatment is imperative, and tests that are commonly used in the diagnostic work-up of anaemia, such as haemoglobin (Hb) and serum ferritin, are readily available.^6^ Despite this, identifying and treating iron deficiency anaemia (IDA) can be challenging, partly because ferritin can be elevated in inflammation. Common underlying causes of iron deficiency include dietary insufficiency, malabsorption, and menorrhagia. However, IDA may also be an indication of underlying gastrointestinal malignancy and endoscopic examination may not always detect this in patients.^7^

Despite epidemiological data on anaemia being available on a global scale, the prevalence in the UK is not well described to the best of the authors’ knowledge. The National Institute for Health and Care Excellence (NICE) states that the prevalence of IDA in the UK is around 3% and 8% for adult males and adult females, respectively.^8^ However, sources for these statistics are not obvious. In addition, the few studies that have examined the management of anaemia in the UK have concluded that anaemia was being inadequately investigated.^9^^–^^11^

**Table:** How this fits in

The Global Burden of Disease Study estimates anaemia prevalence in Europe to be 6.0%, but there are no recent studies in the UK to the best of the authors’ knowledge. The estimated prevalence was 4.1% (583 847/14 207 841) (5.1% [363 438/7 121 614] females and 3.1% [220 409/7 086 227] males) in England in 2019 in the current study, but some demographic groups have much higher prevalence. Ferritin testing was not always done in people with microcytic or normocytic anaemia. People with a haemoglobin test indicating anaemia did not always seem to be followed up in primary care.

As part of a programme of research to address the burden of anaemia, this study aims to describe anaemia prevalence, and patterns of Hb and serum ferritin testing in England. These estimates will inform researchers, clinicians, and policymakers so that anaemia and its consequences can be better addressed. The Clinical Practice Research Datalink (CPRD) Aurum database was used in the study; this is a database of anonymised data from UK primary care containing records from 41 million patients registered at 1489 practices.^12^

## Method

The study population consisted of people with acceptable data in CPRD Aurum (February 2022 database)^13^ who were registered in their current practice in 2019 and were born in 2017 or before. The acceptable flag means that the patient was permanently registered, and that the data meet basic quality requirements. In the current study, people recorded as having indeterminate gender were excluded. The year 2019 was chosen because this pre-dates the COVID-19 pandemic, which may have altered typical testing patterns. It was also specified that included patients were eligible for linkage to data on Index of Multiple Deprivation (IMD) meaning that the study population was restricted to people registered at practices in England. The CPRD Aurum pregnancy register was used to identify pregnancy dates in 2019.^14^ Ethnicity was classified into five categories (Asian, Black, Mixed, Other, White),^15^ or missing in people with no recorded ethnicity codes.

For each patient, the number of Hb tests in 2019 was counted, and the lowest Hb value in 2019 was extracted together with the Hb test date. The concomitant value for mean corpuscular volume (MCV) and the ferritin value when one was recorded within the next 0–90 days were extracted. The 90-day window allowed for ferritin tests to be done in response to a low Hb. If there was more than one ferritin value, then the one closest in date to the Hb test was chosen.

World Health Organization (WHO) age-specific cut-offs for Hb were used to assess whether patients had anaemia (130 g/L if male and age ≥15 years; 120 g/L if female and age ≥15 years; 120 g/L if age 12–14 years; 115 g/L if age 5–11 years; 110 g/L if age <5 years; 110 g/L for Hb tests done in pregnancy).^16^ About 29% of pregnancies had no outcome recorded and dates associated with these pregnancies are more likely to be inaccurate.^17^ Based on a sensitivity analysis (Supplementary Table S1) the study nevertheless used the same date-specific Hb cut-off for these pregnancies.

Analyses were done separately by gender. The percentage of people who had at least one Hb test in 2019 and the percentage of those tested who had anaemia were calculated. Anaemia prevalence was estimated from the number of people with anaemia divided by the total number of people (whether or not they received an Hb test). Prevalence was calculated overall, and stratified by IMD, ethnicity, and age group. Prevalence was plotted by 5-year age group for each category of IMD or ethnicity. Confidence intervals for proportions were calculated using the normal approximation.

Patients with anaemia were further categorised according to MCV category (microcytic, <80 fL; normocytic, 80–100 fL, macrocytic, >100 fL). The percentage of these patients with low ferritin, that is, IDA, was calculated. WHO guidelines were used for ferritin concentrations assuming no inflammation (12 µg/L children age <5 years; otherwise 15 µg/L).^18^ In a sensitivity analysis low ferritin was defined as <30 μg/L, to allow for increased ferritin levels where coexisting inflammation may be present.^8^^,^^19^

The study also followed a subpopulation of patients who had an Hb test indicating anaemia in January–March 2019, for a maximum of 1 year. The percentages who had another Hb test 3–6 months after that initial test and the percentages of these that remained below the threshold for anaemia were calculated. The authors also did this for the period 6–12 months after the initial test.

Analyses were undertaken using Stata version 18 software.

## Results

The study population contained 14 207 841 people, with an approximately equal gender ratio (Table 1). Nearly half the females (>3.5 million) were aged 15–49 years old, representing this population during their reproductive years. Approximately 9% (314 046/3 523 355) of these were pregnant in 2019.

**Table 1.** Characteristics of the study population and frequency of Hb testing in 2019

**Characteristic**	**Gender**	

	**Female, *n* (%)**	**Male, *n* (%)**
**Total *N***	7 121 614 (100)	7 086 227 (100)

**Age category**		
1–4 years	331 318 (4.7)	346 202 (4.9)
5–11 years	586 881 (8.2)	615 077 (8.7)
12–14 years	234 626 (3.3)	243 913 (3.4)
15–49 years	3 523 355 (49.5)	3 570 156 (50.4)
50–65 years	1 293 689 (18.2)	1 344 887 (19.0)
>65 years	1 151 745 (16.2)	965 992 (13.6)

**Ethnicity category**		
Asian	607 897 (8.5)	621 011 (8.8)
Black	303 876 (4.3)	297 025 (4.2)
Mixed	141 484 (2.0)	132 185 (1.9)
Other	116 708 (1.6)	124 376 (1.8)
White	4 831 857 (67.8)	4 513 732 (63.7)
Missing	1 119 792 (15.7)	1 397 898 (19.7)

**Quintile of IMD**		
1 (Least deprived)	1 420 788 (20.0)	1 380 397 (19.5)
2	1 425 495 (20.0)	1 401 778 (19.8)
3	1 398 949 (19.6)	1 385 188 (19.5)
4	1 501 449 (21.1)	1 513 539 (21.4)
5 (Most deprived)	1 368 704 (19.2)	1 398 898 (19.7)
Missing	6229 (0.1)	6427 (0.1)

**Pregnancy in 2019**		
No pregnancy	6 806 745 (95.6)	7 086 227 (100.0)
Pregnancy	314 869 (4.4)	0 (0.0)

**Number of Hb tests in 2019**		
0	5 051 013 (70.9)	5 627 588 (79.4)
1	1 441 976 (20.2)	1 053 253 (14.9)
2	406 591 (5.7)	261 590 (3.7)
3	124 600 (1.7)	78 807 (1.1)
4	46 167 (0.6)	30 841 (0.4)
≥5	51 267 (0.7)	34 148 (0.5)

*Hb = haemoglobin. IMD = Index of Multiple Deprivation.*

### Hb

Anaemia prevalence was 5.1% (363 438/7 121 614) in females and 3.1% (220 409/7 086 227) in males (4.1% [583 847/14 207 841] overall). Hb tests were recorded in 29.1% (2 070 601/7 121 614) of females and 20.6% (1 458 639/7 086 227) of males, but only 8.8% (628 625/7 121 614) and 5.7% (405 386/7 086 227), respectively, had >1 test. The relative frequency of Hb testing increased with age in both sexes (Table 2, Supplementary Table S2). Anaemia was most common in people aged >65 years. Of those tested in this age group, 25.3% (158 628/627 361) of females and 29.6% (155 279/524 797) of males had low Hb, giving an anaemia prevalence of 13.8% (158 628/1 151 745) in females and 16.1% (155 279/965 992) in males. Among the 15–49-year-olds, the proportion of females tested was double that of males (25.0% [882 444/3 523 355] and 12.5% [447 557/3 570 156], respectively). Furthermore, in this age group, more tests indicated anaemia in females than in males, resulting in prevalences of 3.9% (138 216/3 523 355) of females and 0.5% (19 224/3 570 156) of males.

**Table 2.** Prevalence of Hb testing in the study population in 2019 and of test results below the threshold for anaemia

**Characteristic**	**Total *N***	**At least one Hb test, *n* (%)**	**At least one Hb test below anaemia threshold, *n* (% of tested)**	**Prevalence of anaemia, %^a^**
**Female**				
All	7 121 614	2 070 601 (29.1)	363 438 (17.6)	5.1
Age category				
1–4 years	331 318	7640 (2.3)	992 (13.0)	0.3
5–11 years	586 881	24 088 (4.1)	1949 (8.1)	0.3
12–14 years	234 626	25 327 (10.8)	3790 (15.0)	1.6
15–49 years	3 523 355	882 444 (25.0)	138 216 (15.7)	3.9
50–65 years	1 293 689	503 741 (38.9)	59 863 (11.9)	4.6
>65 years	1 151 745	627 361 (54.5)	158 628 (25.3)	13.8
Ethnicity category				
Asian	607 897	186 723 (30.7)	56 835 (30.4)	9.3
Black	303 876	89 069 (29.3)	30 422 (34.2)	10.0
Mixed	141 484	30 825 (21.8)	6760 (21.9)	4.8
Other	116 708	27 879 (23.9)	5806 (20.8)	5.0
White	4 831 857	1 513 065 (31.3)	225 222 (14.9)	4.7
Missing	1 119 792	223 040 (19.9)	38 393 (17.2)	3.4
Quintile of IMD^b^				
1 (Least deprived)	1 420 788	407 477 (28.7)	60 677 (14.9)	4.3
2	1 425 495	414 915 (29.1)	65 182 (15.7)	4.6
3	1 398 949	405 608 (29.0)	69 666 (17.2)	5.0
4	1 501 449	427 443 (28.5)	81 879 (19.2)	5.5
5 (Most deprived)	1 368 704	413 672 (30.2)	85 816 (20.7)	6.3

**Male**				
All	7 086 227	1 458 639 (20.6)	220 409 (15.1)	3.1
Age category				
1–4 years	346 202	9384 (2.7)	1409 (15.0)	0.4
5–11 years	615 077	22 206 (3.6)	1644 (7.4)	0.3
12–14 years	243 913	14 112 (5.8)	674 (4.8)	0.3
15–49 years	3 570 156	447 557 (12.5)	19 224 (4.3)	0.5
50–65 years	1 344 887	440 583 (32.8)	42 179 (9.6)	3.1
>65 years	965 992	524 797 (54.3)	155 279 (29.6)	16.1
Ethnicity category				
Asian	621 011	128 058 (20.6)	18 983 (14.8)	3.1
Black	297 025	55 979 (18.8)	10 792 (19.3)	3.6
Mixed	132 185	17 212 (13.0)	2164 (12.6)	1.6
Other	124 376	18 522 (14.9)	1853 (10.0)	1.5
White	4 513 732	1 069 031 (23.7)	161 823 (15.1)	3.6
Missing	1 397 898	169 837 (12.1)	24 794 (14.6)	1.8
Quintile of IMD^b^				
1 (Least deprived)	1 380 397	291 709 (21.1)	43 237 (14.8)	3.1
2	1 401 778	298 671 (21.3)	45 066 (15.1)	3.2
3	1 385 188	286 461 (20.7)	43 296 (15.1)	3.1
4	1 513 539	296 128 (19.6)	44 656 (15.1)	3.0
5 (Most deprived)	1 398 898	284 603 (20.3)	44 010 (15.5)	3.1

a *Anaemia prevalence is the number of people with Hb below the threshold for anaemia divided by total number of people in the stratum (*N*).*

b *Excludes people with missing IMD. Hb = haemoglobin. IMD = Index of Multiple Deprivation.*

**Figure 1.**
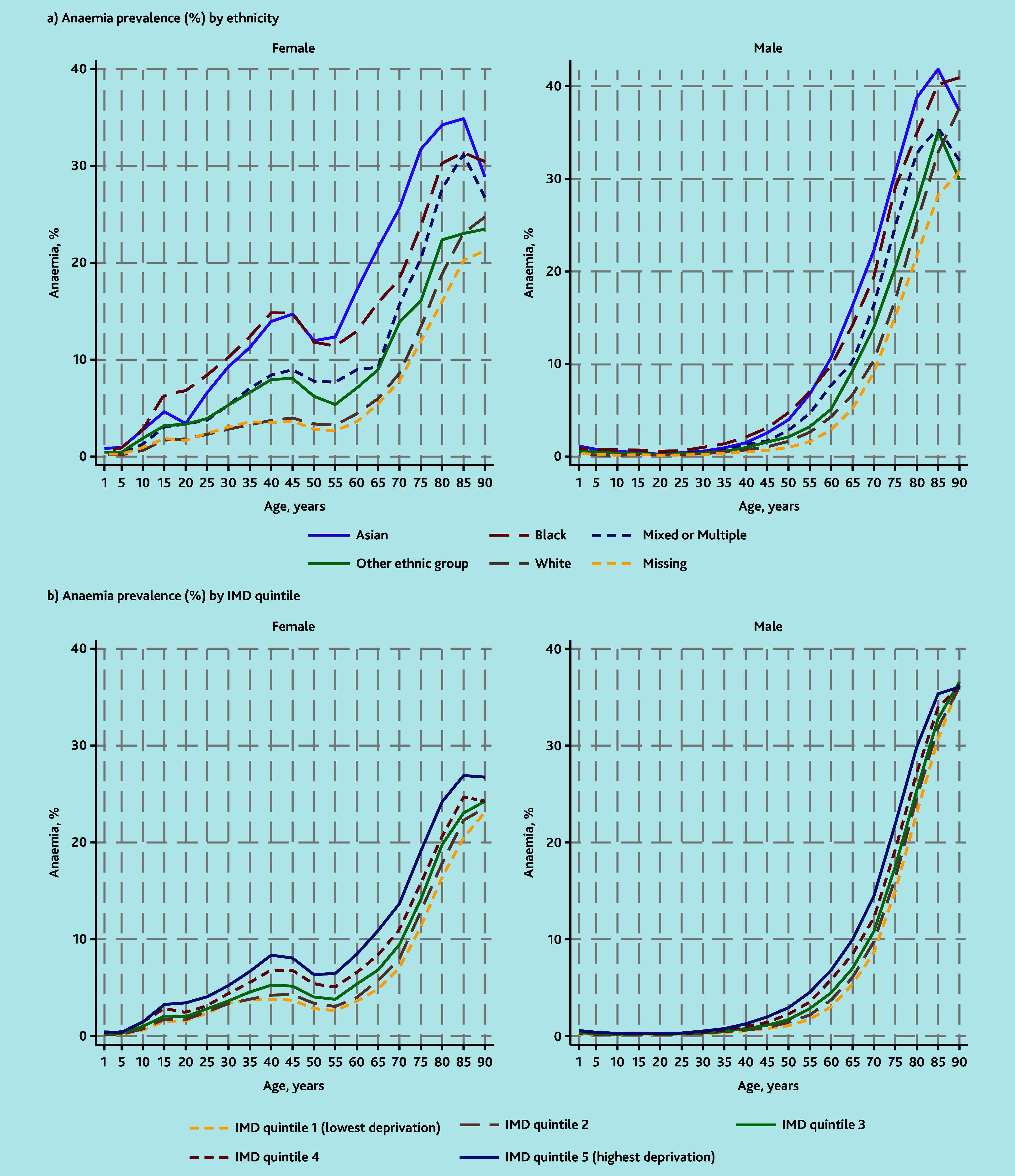
Anaemia prevalence by a) ethnicity and b) quintile of IMD. Anaemia prevalence was calculated for each 5-year age group as the number of people with haemoglobin below the age-and gender-specific threshold in the category, divided by the total in the population for that category. Values are plotted at the lower end of each 5-year age group category and interpolated by straight lines. People with missing IMD were excluded from (b). IMD = Index of Multiple Deprivation.

Variation in anaemia prevalence between females of different ethnicities was evident from late childhood (Figure 1). Similar percentages (approximately 30%) of Asian, Black, and White females received tests in 2019, but within those twice as many Asian and Black females had Hb values below the threshold for anaemia compared with White females (Table 2, Supplementary Table S2). Anaemia prevalence was therefore higher in Asian and Black females (9.3% [56 835/607 897] and 10.1% [30 422/303 876], respectively) compared with White females (4.7% [225 222/4 831 857]). Prevalence was higher in Black compared with Asian females up until about age 45 years, but the reverse was true in older females. Anaemia prevalence in females also increased with social deprivation (Table 2, Supplementary Table S2), and differences were again evident from around late childhood (Figure 1).

Asian and Black males, and males living in areas with higher deprivation, also had higher prevalence of anaemia (Table 2, Supplementary Table S2), although differences were not obvious until after about age 35 years (Figure 1).

### MCV

**Figure 2.**
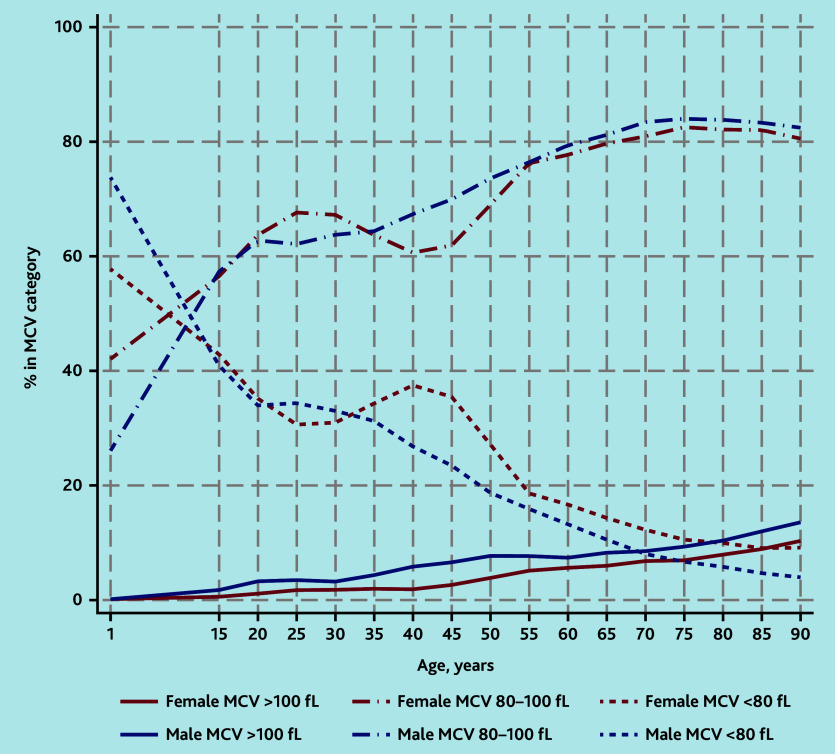
Classification of anaemia by category of MCV. Percentages in each MCV category were calculated separately by age group and gender. Age groups are 1–14 years, and then 5-year age groups from age 15 years. Values are plotted at the lower end of each 5-year age group category and interpolated by straight lines. MCV = mean corpuscular volume.

Some 565 098 (96.8%) of the 583 847 people with anaemia had an MCV result on the same day as their Hb test. The percentage with microcytic anaemia (<80 fL), often indicative of iron deficiency, decreased with older age. Microcytic anaemia accounted for a higher proportion of cases of anaemia in females than in males from around age 35 years (Figure 2). However, when pregnant females, who tend to have higher MCV measurements in pregnancy, were excluded, microcytic anaemia accounted for relatively more cases of anaemia in females from around age 15 years (Supplementary Figure S1).

Macrocytic anaemia (>100 fL) increased gradually with age up to 7.7% (12 255/158 511) (females) and 10.0% (15 511/154 457) (males) of all anaemia cases in those aged >65 years with an MCV result (Figure 2).

### Ferritin

Within people with anaemia and an MCV result, 266 665/565 098 (47.2%) had a ferritin value within 90 days. Of these ferritin results, 73.8% (196 796/266 665) were on the same day as the Hb and MCV. NICE recommends ferritin testing to confirm IDA in all people with an MCV of <95 fL.^8^ Only 67.3% (52 936/78 648) and 45.9% (117 706/256 576) of females with microcytic or normocytic anaemia, respectively, had ferritin tested (Table 3, Supplementary Table S3). Equivalent percentages in males were lower: 57.5% (13 666/23 762) and 40.0% (67 823/169 585), respectively. However, nearly 40% of both males (7630/19 184) and females (6904/17 343) with macrocytic anaemia still had a ferritin value recorded.

**Table 3.** Ferritin testing in people with anaemia by category of MCV^a^

**Age category**	**Total *N***	**Tested for ferritin, *n* (%)**	**Ferritin below WHO age-specific cut-offs, *n* (%)^b^**	**Ferritin <30 µg/L, *n* (%)^b^**
**Female**				
MCV <80 fL				
All ages	78 648	52 936 (67.3)	33 611 (63.5)	41 687 (78.7)
1–14 years	3691	2594 (70.3)	1518 (58.5)	2013 (77.6)
15–49 years	46 401	32 954 (71.0)	23 250 (70.6)	27 777 (84.3)
50–65 years	11 957	7376 (61.7)	3997 (54.2)	4989 (67.6)
>65 years	16 599	10 012 (60.3)	4846 (48.4)	6908 (69.0)
MCV 80–100 fL				
All ages	256 576	117 706 (45.9)	32 705 (27.8)	57 791 (49.1)
1–14 years	2688	1642 (61.1)	762 (46.4)	1193 (72.7)
15–49 years	84 523	45 976 (54.4)	21 175 (46.1)	32 872 (71.5)
50–65 years	43 358	19 085 (44.0)	4202 (22.0)	7621 (39.9)
>65 years	126 007	51 003 (40.5)	6566 (12.9)	16 105 (31.6)
MCV >100 fL				
All ages	17 343	6904 (39.8)	185 (2.7)	790 (11.4)

**Male**				
MCV <80 fL				
All ages	23 762	13 666 (57.5)	5341 (39.1)	7872 (57.6)
1–14 years	2498	1572 (62.9)	611 (38.9)	1046 (66.5)
15–49 years	5227	2972 (56.9)	1063 (35.8)	1322 (44.5)
50–65 years	6087	3395 (55.8)	1467 (43.2)	1981 (58.4)
>65 years	9950	5727 (57.6)	2200 (38.4)	3523 (61.5)
MCV 80–100 fL				
All ages	169 585	67 823 (40.0)	4608 (6.8)	13 279 (19.6)
1–14 years	883	453 (51.3)	44 (9.7)	189 (41.7)
15–49 years	11 909	4786 (40.2)	517 (10.8)	1027 (21.5)
50–65 years	31 178	12 708 (40.8)	1207 (9.5)	2893 (22.8)
>65 years	125 615	49 876 (39.7)	2840 (5.7)	9170 (18.4)
MCV >100 fL				
All ages	19 184	7630 (39.8)	50 (0.7)	314 (4.1)

a *There were 10 871 females and 7878 males who had anaemia but were missing MCV and so are not included in the table.*

b *Ferritin below WHO age-specific cut-offs,* n *(%) and Ferritin <30 μg/L,* n *(%) are %s of those tested for ferritin. MCV = mean corpuscular volume. WHO = World Health Organization.*

In females with microcytic or normocytic anaemia who were tested for ferritin, 63.5% (33 611/52 936) and 27.8% (32 705/117 706), respectively, had IDA (Table 3, Supplementary Table S3).

When the higher threshold of 30 fL was applied to allow for inflammation, the percentages of females potentially with IDA were 78.7% (41 687/52 936) and 49.1% (57 791/117 706), respectively.

In males with microcytic or normocytic anaemia only 39.1% (5341/13 666) and 6.8% (4608/67 823), respectively, of those tested had IDA. Applying the higher ferritin threshold then 57.6% (7872/13 666) and 19.6% (13 279/67 823), respectively, may have had IDA.

Females aged 15–49 years made up the majority of people with confirmed IDA (Table 3, Supplementary Table S3). Most people aged >65 years with anaemia had normocytic anaemia. Only 12.9% (6566/51 003) (or 31.6% [16 105/51 003] allowing for inflammation) of the females in this age group who were tested for ferritin and 5.7% (2840/49 876) (or 18.4% [9170/49 876] allowing for inflammation) of the males had IDA.

### Follow-up Hb tests

Overall, 34.8% (40 659/116 990) of females with anaemia had another Hb test in the following 3–6 months (Table 4). Percentages retested increased with age from 17.8% (332/1867) in children to 41.3% (22 600/54 750) in those >65 years old. The percentage with a retest within 6–12 months was slightly higher but trends were similar. Similar patterns were seen in males.

**Table 4.** Retesting of Hb in people with an Hb below the threshold for anaemia in January to March 2019^a^

**Characteristic**	**Anaemia, *N***	**Retested, *n***	**Retested, % (95% CI)**	**Hb still below anaemia threshold, *n***	**Hb still below anaemia threshold, % (95% CI)**
**Female**					
3–6 months from initial test^b^					
All ages	116 990	40 659	34.8 (34.5 to 35.0)	28 354	69.7 (69.5 to 70.0)
1–14 years	1867	332	17.8 (16.0 to 19.5)	168	50.6 (48.3 to 52.9)
15–49 years	40 922	10 310	25.2 (24.8 to 25.6)	5648	54.8 (54.3 to 55.3)
50–65 years	19 451	7417	38.1 (37.4 to 38.8)	5079	68.5 (67.8 to 69.1)
>65 years	54 750	22 600	41.3 (40.9 to 41.7)	17 459	77.3 (76.9 to 77.6)
6–12 months from initial test^c^					
All ages	112 686	56 358	50.0 (49.7 to 50.3)	37 684	66.9 (66.6 to 67.1)
1–14 years	1834	428	23.3 (21.4 to 25.3)	209	48.8 (46.5 to 51.1)
15–49 years	39 895	14 923	37.4 (36.9 to 37.9)	8162	54.7 (54.2 to 55.2)
50–65 years	19 011	10 324	54.3 (53.6 to 55.0)	6622	64.1 (63.5 to 64.8)
>65 years	51 946	30 683	59.1 (58.6 to 59.5)	22 691	74.0 (73.6 to 74.3)

**Male**					
3–6 months from initial test^b^					
All ages	74 005	27 976	37.8 (37.5 to 38.2)	22 555	80.6 (80.3 to 80.9)
1–14 years	1220	171	14.0 (12.1 to 16.0)	79	46.2 (43.4 to 49.0)
15–49 years	5759	1544	26.8 (25.7 to 28.0)	989	64.1 (62.8 to 65.3)
50–65 years	13 298	4691	35.3 (34.5 to 36.1)	3294	70.2 (69.4 to 71.0)
>65 years	53 728	21 570	40.1 (39.7 to 40.6)	18 193	84.3 (84.0 to 84.7)
6–12 months from initial test^c^					
All ages	70 294	39 136	55.7 (55.3 to 56.0)	30 163	77.1 (76.8 to 77.4)
1–14 years	1189	227	19.1 (16.9 to 21.3)	96	42.3 (39.5 to 45.1)
15–49 years	5612	2154	38.4 (37.1 to 39.7)	1266	58.8 (57.5 to 60.1)
50–65 years	12 895	6821	52.9 (52.0 to 53.8)	4525	66.3 (65.5 to 67.2)
>65 years	50 598	29 934	59.2 (58.7 to 59.6)	24 276	81.1 (80.8 to 81.4)

a *Time periods 3–6 months and 6–12 months after the initial low Hb were considered.*

b *Excludes people who were no longer in the cohort 3 months after the initial low Hb result.*

c *Excludes people who were no longer in the cohort 6 months after the initial low Hb result. CI = confidence interval. Hb = haemoglobin.*

## Discussion

### Summary

The overall anaemia prevalence was 4.1% (583 847/14 207 841) indicating a burden comparable with those of other common disorders, such as diabetes or heart disease. Some population groups had much higher prevalence of anaemia, including older people, people of Black or Asian ethnicity, and those living in areas with high social deprivation. Females of reproductive age also had higher levels of testing and anaemia. Differences between ethnic groups and quintiles of IMD were evident in females of reproductive age as well as in older males and females.

The data in the current study also raise questions about inconsistency in testing. NICE recommends that ferritin should be checked in everyone with an MCV of <95 fL. However, many people with microcytic or normocytic anaemia were missing a corresponding ferritin value. Longitudinal analyses also suggested that Hb tests indicating anaemia are often not followed up.

### Strengths and limitations

The strength of this study is the very large database of real-world data, which allowed a detailed breakdown of anaemia prevalence and testing. The database is approximately representative of the population in age and gender distribution.^12^ However, tests would be conducted for a clinical indication so anaemia prevalence in the current study is for known or suspected anaemia and is likely to be lower than from prevalence surveys. The degree of underestimation of the population prevalence is likely to vary with demographic group. Underestimation is most likely to be in those with mild anaemia symptoms or in those who do not interact with health services.

Full blood counts requested in other healthcare settings may not be incorporated into primary care records. Therefore the study may have missed anaemia in patients receiving care elsewhere, particularly those with poorer health receiving care in secondary or tertiary centres. A further limitation is that in the current study pregnant and non-pregnant females are reported on together as the authors plan to research anaemia in pregnant females separately. Also exact dates for some pregnancies were not available, and additionally physiological changes in pregnancy mean that anaemia is less straightforward to define from laboratory tests.^20^ Overall, the current study may have slightly overestimated anaemia prevalence in females aged 15–49 years.

### Comparison with existing literature

The current study’s overall estimate of anaemia prevalence is slightly lower than the 6.0% from the Global Burden of Disease study.^1^ Higher prevalence of anaemia in older age, in females of reproductive age, in Black and Asian ethnicities, and at higher levels of social deprivation are broadly consistent with current understanding of anaemia on a global scale.^21^^,^^22^

The study found that in the region of 14–16% of people in the >65 years age group had anaemia, which is consistent with a systematic review of studies done in various settings in developed countries and is probably associated with chronic disease and inflammation.^23^ The current findings for this age group are also like those from a study with older primary care patients (>65 years) who had full blood counts in Oxfordshire in 2012–2013,^10^ not only for the prevalence of anaemia but also the degree of incompleteness of ferritin testing, and lack of follow-up for further Hb testing.

One population-based cross-sectional study of European and Asian populations in Newcastle upon Tyne (UK) found anaemia to be more prevalent in females of Asian ethnicity than those of White ethnicity.^24^ The authors of the current study could not find any other UK studies of anaemia by age group and ethnicity. Data from the US National Health and Nutrition Examination Survey reported similarly wide differences in anaemia prevalence in older people of Black ethnicity compared with White,^25^ and between Black and White ethnicities in females of reproductive age.^26^ Causes of differences in anaemia prevalence between ethnicities are likely to be multifactorial including higher rates of menorrhagia in Black females, differences in social deprivation, diet, prevalence of other chronic diseases, and of inherited causes of anaemia.^27^^–^^29^

### Implications for research and practice

This study has highlighted a high burden of anaemia throughout all ages, with public health implications for better understanding the consequences, and for implementing strategies for optimal diagnosis and management.

Differences in anaemia prevalence between ethnicities and levels of social deprivation that vary with age and gender may be because of multiple underlying factors, and disentangling these influences should be a priority for further research.^29^^,^^30^ Investigation of high anaemia prevalence and its management in Black and Asian females of reproductive age is of particular importance given the association of anaemia with poorer pregnancy outcomes, and clinical guidelines already emphasise early detection and treatment of anaemia in pregnancy.^20^ The current results suggest that there should also be emphasis on diagnosing and managing anaemia before pregnancy, particularly in these high-risk groups. The increase in macrocytic anaemia in people aged >65 years may be due, in part, to the increase in disorders such as myelodysplasia,^30^ and monitoring older patients with macrocytic anaemia may help to understand and predict bone marrow disorders.^31^

Around half of people with microcytic or normocytic anaemia had a ferritin test result recorded even though NICE recommends testing in all people with an MCV of <95 fL.^8^ This represents a missed opportunity for identification of IDA and treatment initiation that clinicians can address. Further research using primary care records could tell us whether patients who do not have a ferritin result are also less likely to be prescribed iron and to have a persistent anaemia at 3–12 months. Primary care data and linked secondary care data could be used to evaluate treatment and patient outcomes following an initial diagnosis of anaemia in primary care and how prompt treatment can influence outcomes. For example, patients are often seen in primary care during preparation for surgery, and anaemia is a risk factor for poorer surgical outcomes in tertiary settings.^32^ Most ferritin tests were recorded on the same date as an Hb value; the utility and cost-effectiveness of routinely testing ferritin at the same time as Hb should also be investigated. This could have the added benefit of detecting patients with iron deficiency without anaemia that is increasingly being recognised as a clinical condition requiring treatment.^33^

The current findings also suggest that there may be a longitudinal burden of under-investigated and untreated anaemia over the life course. However, lack of follow-up Hb tests for people with an initial low Hb may not necessarily indicate that there was no intervention as the Hb may have been borderline, symptoms may have improved without treatment, or follow-up may have been in secondary care. Nevertheless, research is needed to further understand why some low Hb results do not appear to be followed up.

In summary, the current data indicate a high prevalence of anaemia in the UK population, with many areas of research needed. Primary care offers the ideal setting to detect and manage anaemia accordingly, which may prevent poor health from occurring downstream. There appears to be suboptimal adherence to published guidelines particularly in relation to performing additional tests (for example, ferritin) when indicated. An implementation-science approach may be required to address this. Predictive models, for example, machine learning algorithms, could be developed to identify at-risk individuals so they can be treated in a timely manner.

## Data

Data can be accessed by application to the CPRD. The authors have uploaded codelists to Github. See: https://github.com/ndpchs-cprd/22-001873-Anaemia/tree/main/Smith/Codelists.

## References

[1] Gardner WM, Razo C, McHugh TA (2023). Prevalence, years lived with disability, and trends in anaemia burden by severity and cause, 1990–2021: findings from the Global Burden of Disease Study 2021. Lancet Haematol.

[2] Cappellini MD, Musallam KM, Taher AT (2020). Iron deficiency anaemia revisited. J Intern Med.

[3] Brittenham GM, Moir-Meyer G, Abuga KM (2023). Biology of anemia: a public health perspective. J Nutr.

[4] Palapar L, Kerse N, Rolleston A (2021). Anaemia and physical and mental health in the very old: an individual participant data meta-analysis of four longitudinal studies of ageing. Age Ageing.

[5] Benson CS, Shah A, Stanworth SJ (2021). The effect of iron deficiency and anaemia on women’s health. Anaesthesia.

[6] Kumar A, Sharma E, Marley A (2022). Iron deficiency anaemia: pathophysiology, assessment, practical management. BMJ Open Gastroenterol.

[7] Stone H, Almilaji O, John C (2022). Original research: the dedicated iron deficiency anaemia clinic: a 15-year experience. Frontline Gastroenterol.

[8] National Institute for Health and Care Excellence (2024). Anaemia — iron deficiency.

[9] Logan ECM, Yates JM, Stewart RM (2002). Investigation and management of iron deficiency anaemia in general practice: a cluster randomised controlled trial of a simple management prompt. Postgrad Med J.

[10] McCartney D, Shine B, Hay D, Lasserson DS (2017). The evaluation of anaemia in an older primary care population: retrospective population-based study. BJGP Open.

[11] Hamid M, Naz A, Alawattegama LH, Steed H (2021). The prevalence of anaemia in a district general hospital in the United Kingdom. Cureus.

[12] Wolf A, Dedman D, Campbell J (2019). Data resource profile: Clinical Practice Research Datalink (CPRD) Aurum. Int J Epidemiol.

[13] Medicines & Healthcare products Regulatory Agency (2022). CPRD Aurum February 2022 dataset.

[14] Campbell J, Shepherd H, Welburn S (2023). Methods to refine and extend a Pregnancy Register in the UK Clinical Practice Research Datalink primary care databases. Pharmacoepidemiol Drug Saf.

[15] Office for National Statistics (2023). Ethnic group by age and sex, England and Wales: Census 2021.

[16] World Health Organization (2011). Haemoglobin concentrations for the diagnosis of anaemia and assessment of severity.

[17] Campbell J, Bhaskaran K, Thomas S (2022). Investigating the optimal handling of uncertain pregnancy episodes in the CPRD GOLD Pregnancy Register: a methodological study using UK primary care data. BMJ Open.

[18] World Health Organization (2020). WHO guideline on use of ferritin concentrations to assess iron status in individuals and populations.

[19] Snook J, Bhala N, Beales ILP (2021). British Society of Gastroenterology guidelines for the management of iron deficiency anaemia in adults. Gut.

[20] Pavord S, Daru J, Prasannan N (2020). UK guidelines on the management of iron deficiency in pregnancy. Br J Haematol.

[21] Safiri S, Kolahi AA, Noori M (2021). Burden of anemia and its underlying causes in 204 countries and territories, 1990–2019: results from the Global Burden of Disease Study 2019. J Hematol Oncol.

[22] Stauder R, Valent P, Theurl I (2018). Anemia at older age: etiologies, clinical implications, and management. Blood.

[23] Gaskell H, Derry S, Andrew Moore R, McQuay HJ (2008). Prevalence of anaemia in older persons: systematic review. BMC Geriatr.

[24] Fischbacher C, Bhopal R, Patel S (2001). Anaemia in Chinese, South Asian, and European populations in Newcastle upon Tyne: cross sectional study. BMJ.

[25] Guralnik JM, Eisenstaedt RS, Ferrucci L (2004). Prevalence of anemia in persons 65 years and older in the United States: evidence for a high rate of unexplained anemia. Blood.

[26] Gupta PM, Hamner HC, Suchdev PS (2017). Iron status of toddlers, nonpregnant females, and pregnant females in the United States. Am J Clin Nutr.

[27] Chapple A (1998). Iron deficiency anaemia in women of south Asian descent: a qualitative study. Ethn Health.

[28] Hayanga B, Stafford M, Bécares L (2023). Ethnic inequalities in multiple long-term health conditions in the United Kingdom: a systematic review and narrative synthesis. BMC Public Health.

[29] Weyand AC, Mcgann PT (2021). Eliminating race-based reference ranges in haematology: a call to action. Lancet Haematol.

[30] Stauder R, Valent P, Theurl I (2018). Anemia at older age: etiologies, clinical implications, and management. Blood.

[31] Koshiaris C, Van Den Bruel A, Oke JL (2018). Early detection of multiple myeloma in primary care using blood tests: a case–control study in primary care.. Br J Gen Pract.

[32] Fowler AJ, Ahmad T, Abbott TEF (2018). Association of preoperative anaemia with postoperative morbidity and mortality: an observational cohort study in low-, middle-, and high-income countries. Br J Anaesth.

[33] Al-Naseem A, Sallam A, Choudhury S, Thachil J (2021). Iron deficiency without anaemia: a diagnosis that matters. Clin Med (Lond).

